# M2b macrophages protect against doxorubicin induced cardiotoxicity via alternating autophagy in cardiomyocytes

**DOI:** 10.1371/journal.pone.0288422

**Published:** 2023-07-27

**Authors:** Sida Chen, Yang Huang, Suiqing Huang, Zhuoming Zhou, Kaizheng Liu, Jinyu Pan, Zhongkai Wu

**Affiliations:** Department of Cardiac Surgery, The First Affiliated Hospital, Sun Yat-sen University, Guangzhou, Guangdong, China; Xiangtan University, CHINA

## Abstract

**Objective:**

Doxorubicin (DOX) is an anthracycline antibiotic which is widely used for the treatment of various cancers, while the dose-related cardiotoxicity limits its potential therapeutic application. The underlying mechanism of DOX induced cardiotoxicity is complex and remains elusive. Our previous studies have shown that M2b macrophage plays an important role in reducing inflammation due to ischemic reperfusion injury in the myocardium. The purpose of this study was to investigate the potential protective role of M2b macrophages in DOX induced cardiotoxicity.

**Methods:**

In vivo, we conducted DOX induced cardiac injury in C57BL/6 mice and treated them with M2b macrophages. Then, the mice were examined by echocardiography. The heart specimens were harvested for histological examination, transmission electron microscope analysis, and autophagy molecules evaluation. In vitro, HL-1 cardiac cell lines treated with DOX were cocultured with or without M2b macrophages. Then, Autophagy related genes and protein expression were assessed by real-time quantitative PCR and western blot; cell proliferation was assessed by cell counting kit-8.

**Results:**

We found that M2b macrophages can improve cardiac function and alleviate cardiac injury in DOX induced cardiac injury mice. M2b macrophages can enhance cardiac autophagy levels both in vivo and in vitro in DOX induced cardiac injury model. In addition, this protective effect can be blocked by an autophagy inhibitor.

**Conclusion:**

Our study shows that M2b macrophages can help attenuate the DOX induced cardiotoxicity by regulating the autophagy level of cardiomyocytes.

## Introduction

Doxorubicin (DXR or Adriamycin) is a commonly utilized chemotherapeutic agent. However, the dose-dependent cardiotoxicity leading to irreversible or even fatal congestive heart failure, has limited the potential antitumor efficiency of this drug [1]. Following DOX chemotherapy, the development of cardiomyopathy adversely affects cancer survivors’ long-term cardiac prognosis and significantly restricts their ability to receive treatment for recurrent or refractory disease [2]. Currently, no effective strategy can prevent DOX-related cardiomyopathy in cancer patients and survivors, despite the prevalence and seriousness of DOX-induced cardiac dysfunction. Therefore, to develop cardioprotective medicines, a thorough understanding of the molecular mechanisms behind DOX cardiotoxicity is necessary [3, 4].

The etiology of doxorubicin (DXR)-induced cardiac injury is multifaceted. Accumulating evidence suggests that DXR can induce cardiac injury through multiple mechanisms, including excessive production of reactive oxygen species (ROS), mitochondrial injury, and dysregulated autophagy. For example, DXR has been shown to increase the generation of reactive oxygen species (ROS) in cardiomyocytes. This oxidative stress can lead to oxidative damage to cellular components, such as lipids, proteins, and DNA, thereby compromising the structural and functional integrity of the cardiomyocytes [5]. Altered level of autophagy could lead to DOX-induced cardiac injury, but the underlying mechanism remains uncertain. Autophagy is a highly conserved process which can help to maintain cell homeostasis [6]. The role of autophagy in myocardium protection is contradictory, on the one hand, autophagy is a cellular quality control mechanism that help the clearance of abnormal proteins, malfunctioning organelles or pathogens [7], on the other hand, excessive autophagy may induce apoptosis and shorten the life of cardiomyocytes [8]. However, previous studies have shown conflicting interpretations on dox-induced autophagy, while some research show upregulation and some others show deregulation [9].

The macrophages within heart tissue can play an important role in maintaining the hemostasis of cardiomyocytes including facilitation of electrical conduction in the atrioventricular node, preventing fibrosis, and eliminating the injured mitochondria [10–12]. Furthermore, a recent study by Hanwen Zhang from Nanjing Medical University in 2020 revealed an intriguing finding regarding the role of resident cardiac macrophages in doxorubicin-induced cardiac injury. Zhang demonstrated that administration of doxorubicin can mobilize and stimulate self-renewal of these resident cardiac macrophages. This observation sheds light on the potential involvement of macrophages in the pathogenesis and response to doxorubicin-induced cardiotoxicity. The restoration of resident macrophage during DOX-induced cardiac myopathy development can act as a brake of DOX-induced cardiac myopathy [13]. This effect can protect against adverse cardiac remodeling. This phenomenon indicates that specific population of macrophage can help the heart overcome multiple crisis. Looking into the underlying mechanism of these groups of macrophages may inspire researchers to exploit new cardiac protective target. One of the most discussed potential mechanisms is autophagy.

Our previous studies have shown that M2b macrophage can suppress the apoptosis and fibrosis of cardiomyocyte and pulmonary artery smooth muscle cells [14, 15]. But the mechanism of how macrophage can influence the cardiomyocyte remains elusive [16]. In this study, we analyzed the cardiac protective role of M2b macrophage injection in DOX induced cardiac injury model and found that M2b macrophage can protect from DOX induced cardiac injury by regulating the autophagy level in cardiomyocyte. Therefore, this study may provide new understanding of the autophagic process in DOX-induced cardiac injury and innovative aspects to developing cardioprotective targets.

## Materials and methods

### Cell culture and treatment

Atrial-derived HL-1 cardiomyocytes were purchased from Sigma company and grown in DMEM supplemented with 10% fetal bovine serum, glutamine (2mM), penicillin (100U/ml), streptomycin (100 mg/ml), and pyruvate (1 mM) in humidified air (5% CO2) at 37°C. HL-1 cell at 60–70% density was used for experimental treatment. The cells were treated with DOX (MedChemExpress, China) at indicated concentrations for 9 h, and then cocultured with M2b macrophage supernatant for 24h.

### Experimental animals and DOX administration

A total of 35 C57BL/6 mice (male, 6–8 weeks old, weighing 18-20g) were obtained from the Sun Yat-sen University Laboratory Animal center. To minimize the influence of gender differences, male mice were exclusively included in the study. The mice were maintained on a 12-hour light-dark cycle at a constant temperature of 22±°C with ad libitum access to food and tap water. The standard laboratory chow did not contain any substances that were expected to affect the experimental results. All animal experiments were conducted in compliance with the NIH guidelines (Guide for the Care and Use of Laboratory Animals) and approved by the Institutional Animal Care and Use Committee of Sun Yat-sen University. Animal health and behavior were monitored everyday. Fifteen mice were sacrificed and harvested macrophages, the other 20 mice were randomly assigned to 4 groups each containing five animals, as follows:1) control group, which was intraperitoneally injected with PBS; 2) doxorubicin (DOX) group, intraperitoneally injected with 20mg/kg DOX; 3) DOX+M2b macrophage group, which was intraperitoneally injected with DOX, and 1 million M2b were injected through the caudal vein after DOX injection.; 4) control+M2b group, which was intraperitoneally injected and 1 million M2b macrophages were injected through the caudal vein after DOX injection. The criteria used to determine the endpoint include factors such as the animal’s overall health, pain and suffering, and the likelihood of recovery or improvement. Additionally, ethical considerations such as the prevention of unnecessary suffering are also taken into account. When animals in experiments reached the predetermined endpoint criteria, they were observed for an hour before euthanasia is carried out. The sample size was based on prior studies from Dinender K Singla [17], similar group sizes were used for comparable experiments. The Ethics Committee for the Institutional Animal Use and Care Committee of Sun Yat-sen University, approved the experimental protocols for studies The therapeutic effect of M2b macrophages for DOX-induced cardiac injury (project title: Approval no. 2023000689; Date of approval: Jan 20, 2022).

### Echocardiography and hemodynamic analysis

Mice were anaesthetized with 1.5% isoflurane/oxygen, and cardiac function was assessed using transthoracic echocardiography (VisualSonics system, Toronto, Ontario, Canada), which was performed at the end of the 2-h reperfusion. We performed M-mode and two-dimensional echocardiography to evaluate cardiac parameters, including left ventricular (LV) end-diastolic dimension, wall thickness, LV fractional shortening, and ejection fraction.

### LDH and CK-MB measurements

Serum was obtained from blood by centrifugation at 4000 rpm for 20 min. The levels of lactate dehydrogenase (LDH) and creatine kinase isoenzyme (CK-MB) were measured using an LDH ELISA kit (FineTest) and a CK-MB ELISA kit (Dogesce), respectively.

### Hematoxylin-eosin (H&E) staining

After the mice were sacrificed, the myocardial tissues were separated and fixed in 10% neutral buffered formalin for 24h. Afterwards, the samples were embedded in paraffin and cut into 3-μm sections. The histological sections were then stained with hematoxylin and eosin (H&E) and visualized using a Leica DM3000 biological microscope (Leica, Wetzlar, Germany).

### Transmission electron microscopy

Transmission electron microscopy (TEM) was employed to examine the ultrastructure of myocardial tissues. Briefly, left ventricular tissues from mice were rapidly sectioned into 1 mm cubes and fixed in 2.5% glutaraldehyde at 4°C overnight. Afterwards, the specimens were postfixed with 1% buffered osmium tetroxide and dehydrated in a graded acetone series. The tissue sections were subsequently embedded in epoxy resin and sectioned. Ultrathin sections (70 nm) were obtained and stained with uranyl acetate/lead citrate followed by observation under electron microscopy.

### Isolation and in vitro polarization of macrophages

Mice used for macrophage harvesting were euthanized by cervical dislocation. Bone marrow was collected from femurs and cultured in complete Roswell Park Memorial Institute (RPMI) 1640 medium (Gibco, Grand Island, NY, USA) at 37°C with 5% CO2. For the first 3 days, cells were cultured in RPMI 1640, followed by Dulbecco’s modified Eagle’s medium (DMEM; Gibco) for the subsequent three days to generate mature bone marrow-derived macrophages (BMDMs). Both RPMI 1640 and DMEM had been supplemented with 10% fetal bovine serum (Gibco) and 10ng/ml macrophage colony stimulating factor (PeproTech, Rocky hill, NJ, USA). On day 6, BMDMs were replated in 24-well plates (Corning) and induced to differentiate into M1 macrophages by the addition of 1μg/ml lipopolysaccharide (LPS; Sigma Aldrich, St. Louis, MO, USA), into M2a macrophages with 20ng/ml IL-4 (PeproTech) and 20 ng/ml IL-13 (PeproTech), into M2b macrophages with 50μg/ml IgG (Sigma Aldrich) and 100 ng/ml LPS (Sigma Aldrich), or into M2c macrophages with 20ng/ml IL-10 (PeproTech). After 24 hours of incubation, cells had been harvested to research macrophage markers or re-plated and cultured with clean DMEM without stimulation for some other 24 hours. The cell-unfastened supernatants have been gathered at 24 hours and used for coculture with cardiomyocytes.

### Western blot analysis

The cellular protein was extracted utilizing the RIPA lysis buffer (Beyotime Biotechnology, China) supplemented with a cocktail of protease and phosphorylase inhibitors (Merck Millipore, Billerica, MA, USA). A Bio-Rad protein assay kit (Bio-Rad Laboratories, CA, USA) was used to determine protein concentration. SDS-PAGEs of 10–12% were used to fractionate the whole lysate, which was later transferred to PVDF membranes (Merck Millipore). After blocked with 5% BSA for 1 h, the membranes were incubated overnight at 4°C with primary antibodies against LC3, ATG7, mTOR, phospho-mTOR, SQSTM1/p62 (all obtained from Cell Signaling Technology, USA) and GAPDH (Proteintech, Rosemont, IL, USA). On the following day, the membranes were exposed to the appropriate peroxidase-conjugated secondary antibodies (SouthernBiotech, Birmingham, AL, USA) for 1 h. The protein bands were visualized using Western chemiluminescent HRP substrate (Merck Millipore) and ChemiDoc Touch (Bio-Rad Laboratories).

### CCK-8 assay

Cell proliferation was evaluated using a CCK-8 assay (Beyotime Biotechnology, China) in accordance with the manufacturer’s instructions. HL-1 cells were serum starved in serum-free medium for 24 h prior to the proliferation experiment. 1×10^5^ HL-1 cells were seeded into 96-well plates. Following treatment for 24 h, each well was supplemented with fresh serum-free DMEM solution containing 10μl of CCK-8 solution, followed by incubation at 37°C for 1 h in the dark. Analyses were performed with a multi-technology microplate reader (Thermo Fisher Scientific) by measuring the optical density at 450 nm.

### TUNEL assays

DNA fragmentation analysis was carried out by using a TUNEL fluorescein assay kit (Roche Basel, Switzerland). Tissue slides were incubated with TUNEL reaction mixture (terminal deoxynucleotidyl transferase and nucleotide mixture) for 1 h at 37°C in a humidified and dark atmosphere. Following three washes with PBS, the samples were analyzed using a Meta confocal laser scanning microscope (Carl Zeiss LSM 800).

### Statical analysis

Values are presented as the mean ± SEM. Statis analysis was performed using SPSS 22.0 software. The differences between the two groups were compared by two-tailed Student’s t-test. Comparisons among more than two groups were performed using one-way ANOVA with Tukey post hoc test. To determine statistical significance and indicate a difference between groups, we considered a p-value of less than 0.05 as the threshold.

## Results

### M2b macrophage injection attenuates doxorubicin induced acute cardiac dysfunction in vivo

**Fig 1:**
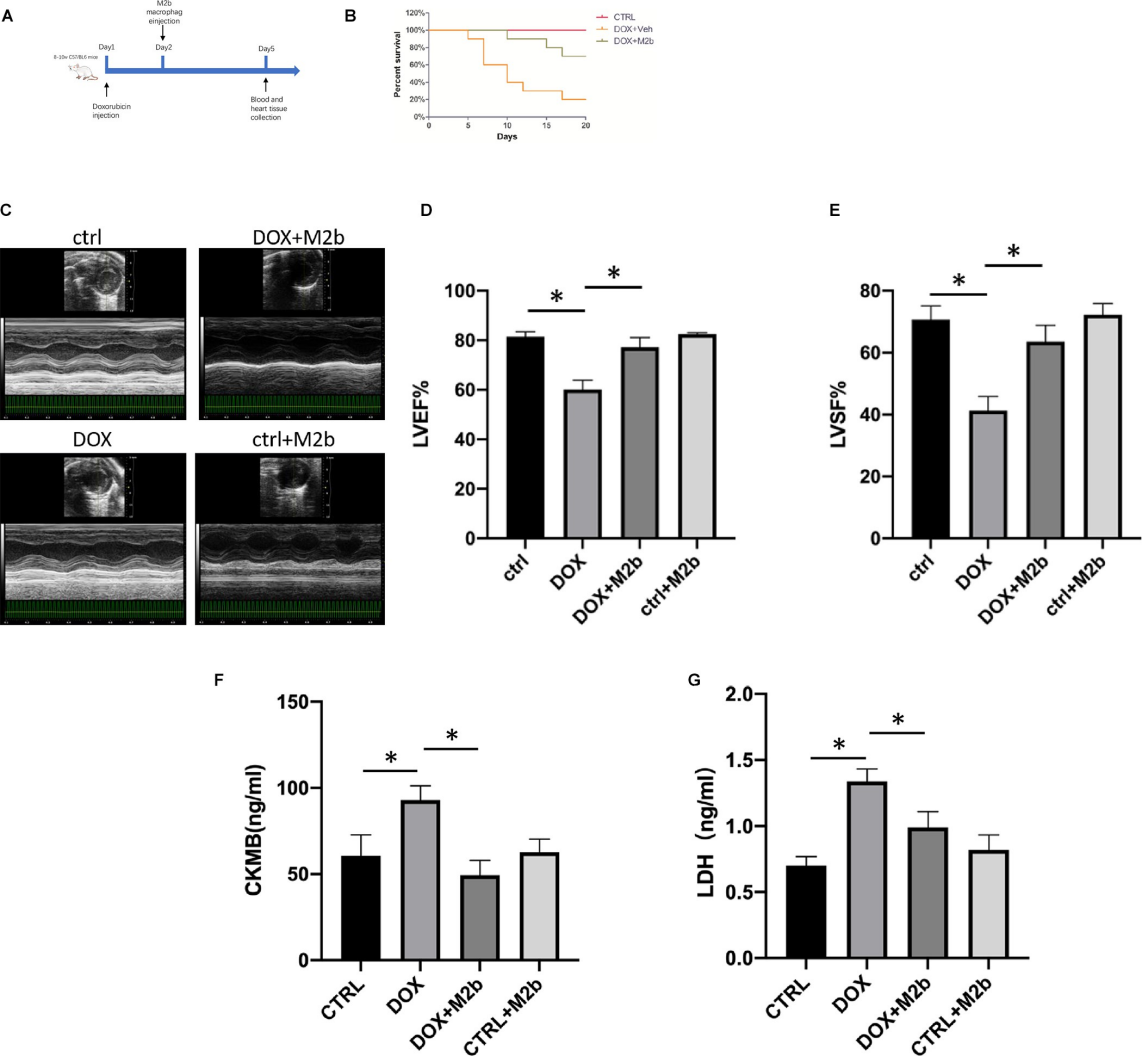
M2b macrophage injection attenuates DOX-induced cardiac injury in mice. (**A**) Schematic of the protocol for animal experiment; (**B**) M2b macrophage improves mortality rate in DOX-induced cardiac injury mice. Kaplan-Meier survival curves in the group (n = 5 for each group); (**C**) Echocardiography of mice; (**D-E**) Left ventricular ejection fraction (LVEF) and fractional shortening (LVFS) in the DOX group(n = 5) were significantly lower than those in the control group(n = 5). Both LVEF and LVFS were higher in the M2b group(n = 5) than those in the DOX group; (**F-G**) The serum CKMB and LDH levels in the DOX group(n = 5) were significantly higher than those in the control group(n = 5). Both CKMB level and LDH level were relatively lower in the M2b group (n = 5) than those in the DOX group. “*” denoting a p-value less than 0.05.

To investigate the effects of external imported M2b macrophages in DOX-induced cardiac injury, 20 C57/BL6 mice were randomly assigned to control group, DOX-treated group, DOX and M2b macrophages treated group, M2b macrophages treated group. After treated with different measures, the cardiac functions of mice were assessed by echocardiography before sacrifice, while the blood and heart tissue were also collected for further examination. Throughout the study, assessments and observations were conducted to monitor the general health status of the animals to determine the experimental endpoint. This included regular visual inspections, as well as recording of any noticeable changes in behavior, appearance, or physiological parameters. The mortality rate is 80% in DOX group and 30% in DOX+M2b group (Fig 1A). We found that M2b macrophage injection decreased the DOX-induced mortality (Fig 1B). In addition, M2b macrophage injection rescued decreased cardiac function, as LVEF and LVFS nearly recovered to normal condition (Fig 1C–1E). In addition, compared with the DOX group, the group injected with M2b macrophages after DOX administration showed a lower level of serum cardiac injury markers including LDH and CK-MB (Fig 1F and 1G). Taken together, M2b macrophage injection demonstrated efficient cardiac protection against DOX-induced cardiac damage.

### M2b macrophage injection attenuates doxorubicin induced acute myocardiocyte injury in vivo

**Fig 2:**
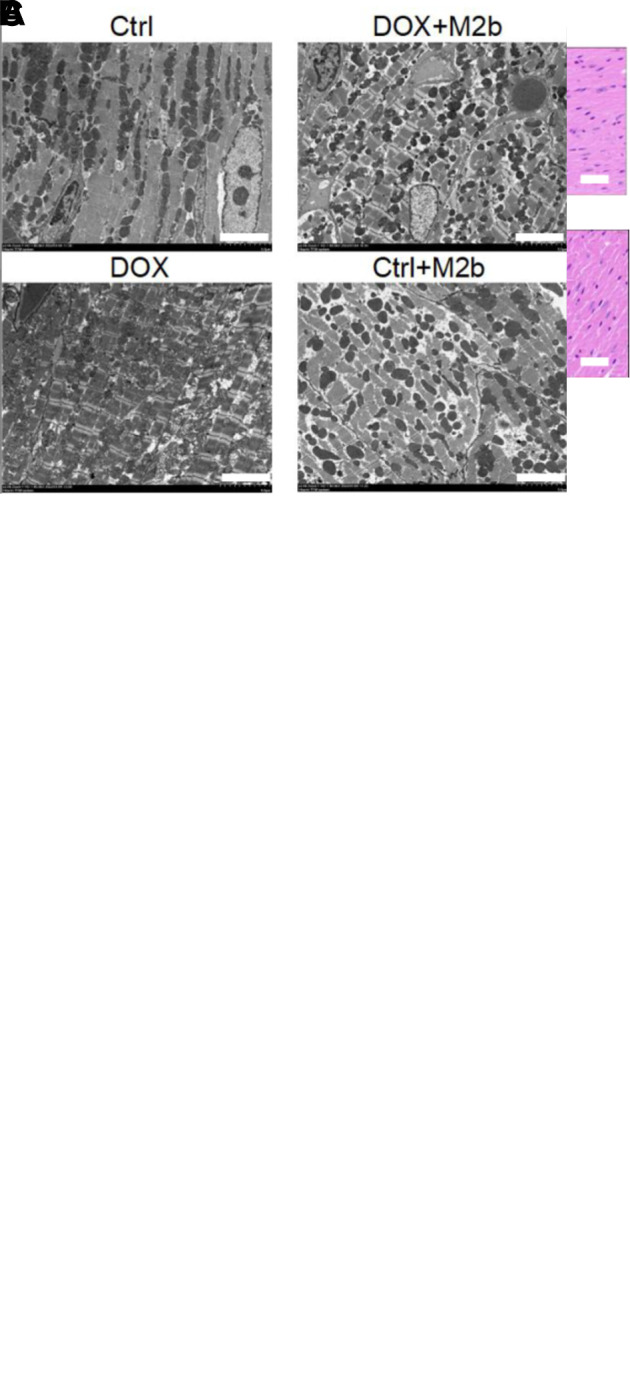
M2b macrophage injection attenuates doxorubicin induced acute myocardiocyte injury in vivo. (**A**) Representative H&E staining micrograph of heart tissue sections(40×). The scale bar is 100 μm in each panel; (**B**) Representative images of the TUNEL assay of heart tissue sections (40×). The scale bar is 100 μm in each panel; (**C**) Representative images of the Transmission electron microscopy of heart tissue sections. The scale bar is 20 μm in each panel.

To explore whether M2b macrophages could attenuate myocardiocyte death after DOX administration, we performed additional evaluations in cardiac tissues. H&E staining revealed that DOX treatment led to more broken myofibers, more vacuolation among fibers, and wider gaps, whereas M2b macrophage injection rescued tissue damage (Fig 2A). The TUNEL assays indicated DOX induced excessive apoptosis compared with the control group, but M2b macrophage injection could significantly reduce cell apoptosis. (Fig 2B). In addition, transmission electron microscope showed that DOX can induce abnormal changes including mitochondrial irregular arrangement, swelling, vacuolated, and disrupted cristae, and these injuries could be attenuated by M2b macrophage injection (Fig 2C). Taken together, these results indicated that M2b macrophages could protect cardiomyocytes in vivo.

### M2b macrophage promotes autophagy in DOX-induced cardiomyopathy in vivo

**Fig 3:**
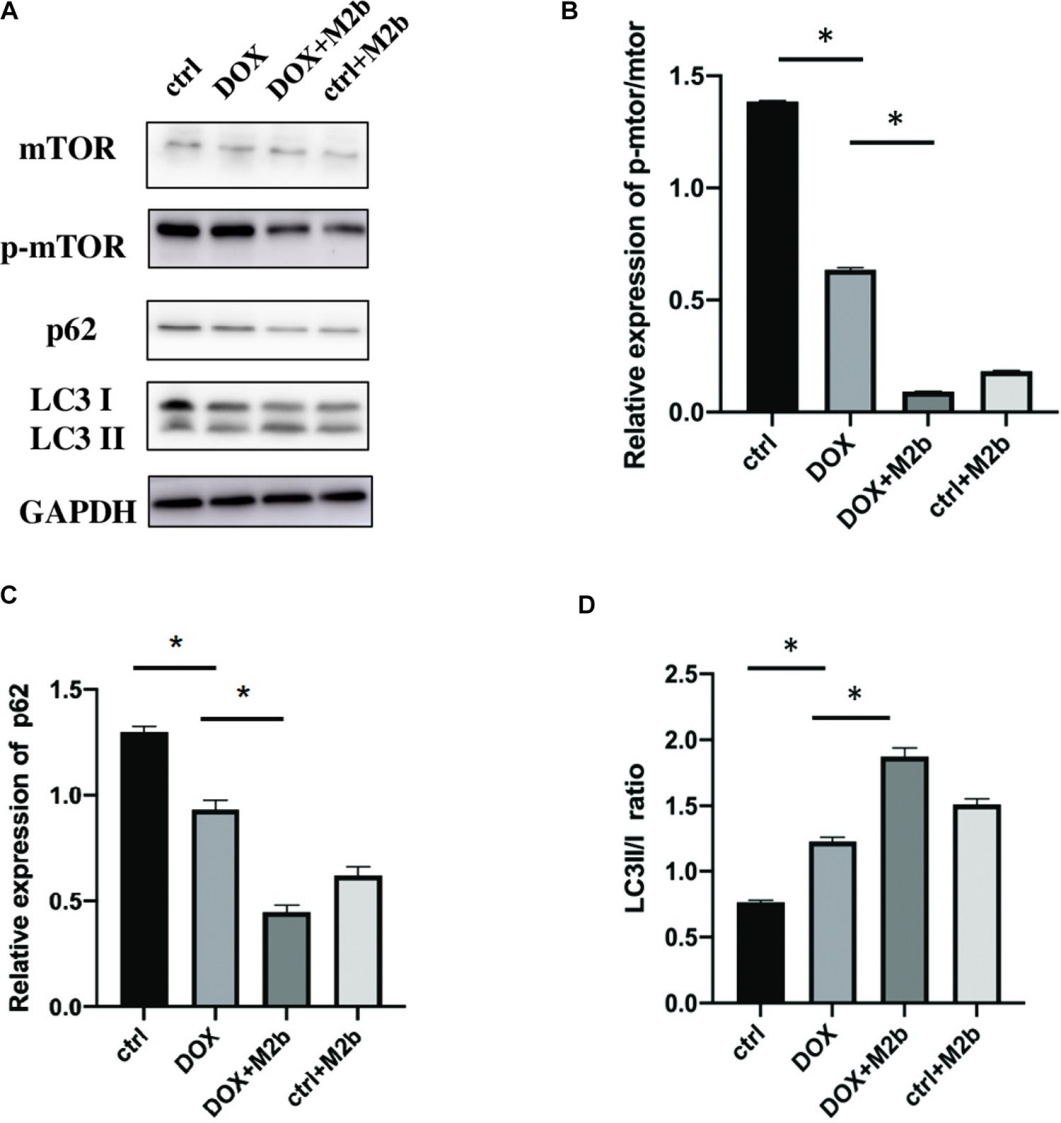
M2b macrophage promotes autophagy in DOX-induced cardiomyopathy in vivo. (**A**) Western blot of mTOR, p-mTOR, P62, LC3 in hearts, full-length blots are presented in S2 Fig; (**B**) Qualification of p-mTOR/mTOR expression in the heart (n = 5 for each group); (**C**) Qualification of P62 expression in the heart (n = 5 for each group); (**D**) Qualification of LC3 expression in the hearts (n = 5 for each group). “*” denoting a p-value less than 0.05.

There are increasing therapeutic strategies for cardio protection targeting autophagy [18, 19]. In a previous study conducted by Yuan Yue, it was found that M2b macrophages play an anti-apoptotic role in cardiomyocytes. This suggests that these specific macrophages have the ability to prevent or inhibit apoptosis, a form of programmed cell death, in cardiomyocytes. This finding implies that M2b macrophages may have a protective effect on cardiomyocytes, potentially contributing to the preservation of cardiac function [14]. Furthermore, studies from Nicolas-Avila show that there is an autophagy regulative role in resident macrophage [12]. Therefore, to investigate whether these roles were related to M2b macrophages mediated protection, in this study, we evaluated the autophagy level among different groups. Western blotting showed that the DOX+M2b group had higher expression of LC3B-II/LC3B-I ratio (Fig 3A). Besides, the DOX+M2b group also had a lower level of P62 and lower phosphorylation level of mTOR (Fig 3A–3C). In summary, these data indicated that M2b macrophage injection could upregulate the autophagy level in vivo during DOX-induced myocardial injury.

### M2b macrophage rescue DOX-induced cardiomyocyte death in an autophagy dependent manner

**Fig 4:**
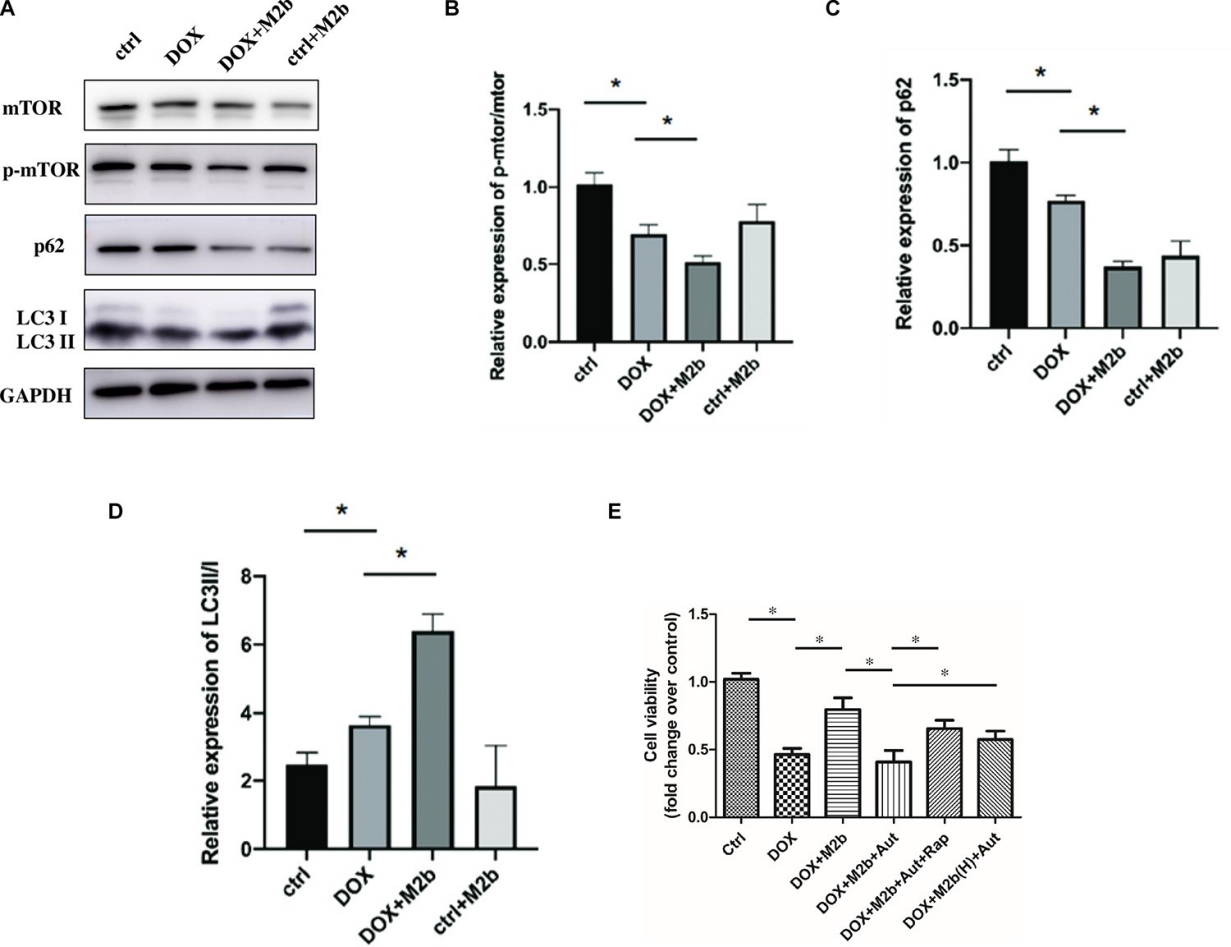
M2b macrophage rescue DOX-induced cardiomyocyte death in an autophagy dependent manner. (**A**) Western blot of mTOR, p-mTOR, P62, LC3 in HL-1 cells, full-length blots are presented in S3 Fig; (**B**) Qualification of p-mTOR/mTOR expression in HL-1 cells (n = 5 for each group); (**C**) Qualification of P62 expression in HL-1 cells (n = 5 for each group); (**D**) Qualification of LC3 expression HL-1 cells (n = 5 for each group); (**E**) CCK-8 assay of HL-1 cells.(5 wells each time for the CCK-8 assay). “*” denoting a p-value less than 0.05. “M2b(H)” indicates the high concentration of M2b supernatant.

To investigate whether M2b macrophages had the same regulative effect on autophagy in vitro, we utilized HL-1 cells as an experimental model. HL-1 cells are a cardiac muscle cell line derived from mouse atrial cardiomyocytes. These cells exhibit several characteristics of primary cardiomyocytes. HL-1 cells are widely used in cardiovascular research as they provide a reliable and reproducible model for studying various aspects of cardiac physiology and pathophysiology. By using HL-1 cells, we aimed to investigate specific mechanisms or phenomena related to cardiomyocytes in a controlled and laboratory-based setting. Western blotting showed that the downregulation of p62 as well as the increasing ratio of LC3B-II/LC3B-I all indicated M2b macrophages could enhance the autophagy flux in DOX-treated HL-1 cells (Fig 4A-4D). The results of the CCK-8 assay indicated that DOX intervention significantly decreased the proliferation capacity of HL-1 cells. Conversely, co-culture with M2b macrophages supernatant reinvigorated proliferation capacity. Nonetheless, this protective effect was thwarted by Autophinib, an inhibitor of autophagy. Furthermore, the addition of rapamycin, a rescue for autophagy, restored cell proliferation. An increased concentration/ of M2b supernatant produced a similar outcome (Fig 4E). In summary, our CCK-8 assay results provide compelling evidence demonstrating the substantial influence of M2b macrophages in enhancing cell survival during DOX-induced cardiac injury in vitro. These effects are likely attributed to their capacity to augment autophagy processes, further highlighting the intricate role of M2b macrophages in promoting cardiomyocyte viability under such pathological conditions.

## Discussion

DOX is one of the most widely used antitumor agents that unfortunately relates to severe acute or dilated cardiotoxicity such as left ventricular dysfunction, dilated cardiomyopathy, and heart failure [20]. Although there are extensive investigations into the molecular mechanisms responsible for DOX cardiotoxicity, a precise understanding remains indetermined [21]. Thus, there is an urgent to uncover the potential mechanisms involved in DOX-induced cardiotoxicity. To the best of our knowledge, this study revealed that M2b macrophages may protect against DOX-induced cardiotoxicity through enhancing autophagy. This observation may contribute to a better understanding of the molecular mechanisms underlying DOX-induced cardiotoxicity.

Previous studies have highlighted the significance of autophagy in the development of DOX-induced cardiotoxicity. For instance, Jian-An Pan et al. demonstrated that Irisin administration effectively mitigates doxorubicin-induced cardiac perivascular fibrosis by modulating autophagy dysfunction in endothelial cells [22] Additionally, Jun Gu et al. found that induction of autophagy by Resveratrol attenuates doxorubicin-induced cardiotoxicity. These studies underscore the crucial role of autophagy regulation in protecting against doxorubicin-induced cardiac damage and provide valuable insights into potential therapeutic strategies for mitigating cardiotoxicity [23].

In physiological conditions, autophagy mediates the selective removal of the damaged organelles [24]. However, the function of autophagy in DOX-induced cardiotoxicity is controversial [25].

On the one hand, the basal level of autophagy under mild stress has been shown to be important, as it preserves myocardial homeostasis and thus maintains normal cardiac function [26]. Previous studies have suggested that boosting autophagy prior to administration may protect against Dox toxicity [27]. Autophagic stimulator, such as Rapamycin, was used to rescue mice from Dox-induced cardiac injury [28]. Starvation for 2 days in mice upregulated autophagy and prevents Dox poisoning [29]. Although stimulation of autophagy prior to Dox appears to be cardioprotective, signaling associated with post-Dox autophagy likely contributes to Dox-induced toxicity and presents a complex and incompletely understood picture. However, inhibition of autophagy initiation and autophagosome formation after DOX administration showed protective effects in DOX-induced cardiac injury. The silencing of Beclin-1 or Atg-5 or the administration of autophagy inhibitors, such as 3-methyladenine, can also protect against DOX-induced cardiac injury [30–33]. In addition, excessive autophagy can consume the majority of the organelle, resulting the loss of functional organelles [34]. The latter suggests that autophagy plays a complex role in pathological processes. Whether autophagy protects against or contributes to DOX-induced cardiotoxicity is still controversial.

In this study, we found that the injection of M2b macrophages reduced DOX- induced cardiac injury. In addition, co-cultured experiments showed that the supernatant of M2b macrophages promotes autophagy in cardiomyocytes, at least in part. This protective effect can be counterbalanced by autophagy inhibitors. The results suggested that M2b macrophages can attenuate DOX-induced cardiac injury by promoting autophagy. Our study provides insight into the impact of M2b macrophages on the control of autophagy levels in cardiomyocytes and potential mechanisms.

At present, there has been minimal research regarding specific types of macrophages such as M2b macrophages. To our knowledge, this is the first study to explore the role of M2b macrophages in DOX-induced cardiac injury. Furthermore, we have not found substantial reports of the involvement of macrophages in autophagy regulation of DOX-induced cardiac injury. M2b macrophages can provide a protective effect on MIRI and reduce inflammation. This effect may be due to the secretion of protective particles. IL-10 is also another potential protective effector because IL-10 was reported to induce autophagy [35].

Although we have revealed the therapeutic role of M2b macrophages in DOX-induced cardiac injury by regulating autophagy levels, there are some limitations in this study. First, the exact molecular mechanisms of the protective effect of M2b macrophages are not clear. The exosome or immune regulatory molecules from M2b macrophages may play a key role in this protective effect. Secondly, we used a single animal model to testify this effect. More experiments should be conducted on transgenic animals to further confirm this protective effect. Third, although the previous study has not reported any side effects from M2b macrophage injection, the potential side effect from immune regulation should be confirmed in further studies.

## Conclusions

In conclusion, this is the first study to explore the protective role of M2b macrophages in DOX-induced cardiac injury and the underlying mechanisms of these effects. The result provides additional insights concerning autophagy regulation by macrophage injection, which may help to develop innovative therapeutic strategies in DOX-induced cardiac injury.

## Supporting information

S1 Fig The identification of M2b macrophages. (A-B) M2b macrophages were subjected to staining for assessment of LIGHT and CD45 expression, followed by analysis through flow cytometry. More than 80% of the cells displayed both LIGHT and CD45 expression (n = 3). (C, D) qRT-PCR was employed to measure the mRNA levels of CCL-1 and IL-10 in macrophages. (*n* = 3 for each group). ***p < 0.001. “M0” indicates the M0 macrophage group, “M2b” indicates the M2b macrophage group.
(PDF)

S2 Fig Images of the uncropped immunoblots shown in Fig 3A. (PDF)

S3 Fig Images of the uncropped immunoblots shown in Fig 4A. (PDF)

## Abbreviations

DOX, DXR: Doxorubicin
ROS: Reactive oxygen species
NIH: National institutes of health
LV: Left ventricular
LDH: Lactate dehydrogenase
CK-MB: Creatine kinase isoenzyme
ELISA: Enzyme linked immunosorbent assay
H&E: Hematoxylin-eosin
TEM: Transmission electron microscopy
RPMI: Roswell Park Memorial Institute
DMEM: Dulbecco’s modified Eagle’s medium
PVDF: Polyvinylidene difluoride
LC3: Microtubule-associated protein light chain 3
ATG: Autophagy-related genes
mTOR: Mechanistic target of rapamycin
SQSTM1: Sequestosome 1
GAPDH: Glyceraldehyde 3-phosphate dehydrogenase
TUNEL: Terminal deoxynucleotidyl transferase dUTP nick end labeling
ANOVA: Analysis of Variance
LVEF: Left ventricular ejection fraction
LVFS: Left ventricular fractional shortening
IL-10: Interleukin-10
