# Supplementary material for: M2b macrophages protect against doxorubicin induced cardiotoxicity via alternating autophagy in cardiomyocytes
Source: PLoS One. 2023 Jul 27;18(7):e0288422. doi: 10.1371/journal.pone.0288422 (PMC10374082; doi:10.1371/journal.pone.0288422)

Materials and Methods for the Supplementary Figure: Identification of M2b Macrophages by Flow Cytometry: Single-cell suspensions were prepared, and then conjugated with rabbit anti-rat LIGHT primary antibody (Abcam) or isotype control (Abcam) followed by Alexa Fluor 488-conjugated donkey anti-rabbit IgG (Invitrogen). The cells were then combined with APC A750-rat CD45 (eBioscience) or isotype control (eBioscience) before being analyzed by flow cytometry using a Beckman Coulter CytoFLEX flow cytometer (Beckman Coulter) and analyzed with FlowJo software. Quantitative Real-Time PCR (qRT-PCR): Total RNA was extracted from tissue homogenates using TRIzol reagent (Invitrogen). Chloroform (200 µl) was used for phase separation, and the RNA was precipitated with 100% isopropanol. After washing twice with 75% ethanol, the RNA was eluted in 30 µl of RNase-free water and the concentration of RNA was measured using a NanoDrop 2000 spectrophotometer (Thermo Fisher Scientific). Reverse transcription was performed on 1000 ng of total RNA per reaction using PrimeScript RT Master Mix (Takara). qRT-PCR was then performed on a Light Cycler 480 system (Roche) using TB Green Premix Ex Taq II (Takara) according to the manufacturer's instructions. The comparative threshold cycle (CT) value for the housekeeping gene GAPDH was used to normalize the loading variations in the PCR. Primers used and their sequences are as follows: CCL-1 Fw: AGAGCCTGCAGTTTCACTCA, Rev: GATCTGTGAGCCTGCATCAGT; IL-10: Fw: GGAGCAGGTGAAGAATGAT, Rev: TCTCGTAGGCTTCTATGCAGTTG; GAPDH Fw: GGTCATCCATGACAACTT, Rev: GGGGCCATCCACAGTCTT.

Figure S1 The identification of M2b macrophages. （A-B）M2b macrophages were stained to assess LIGHT and CD45 expression and were analyzed by flow cytometry. Over 80% of the cells were LIGHT+ CD45+ (n = 3). (C, D) The mRNA levels of CCL-1 and IL-10 were detected by qRT-PCR in macrophages. Data are shown as the mean ± SEM (n = 3 for each group). ***p < 0.001. “M0” indicates the M0 macrophage group, “M2b” indicates the M2b macrophage group.

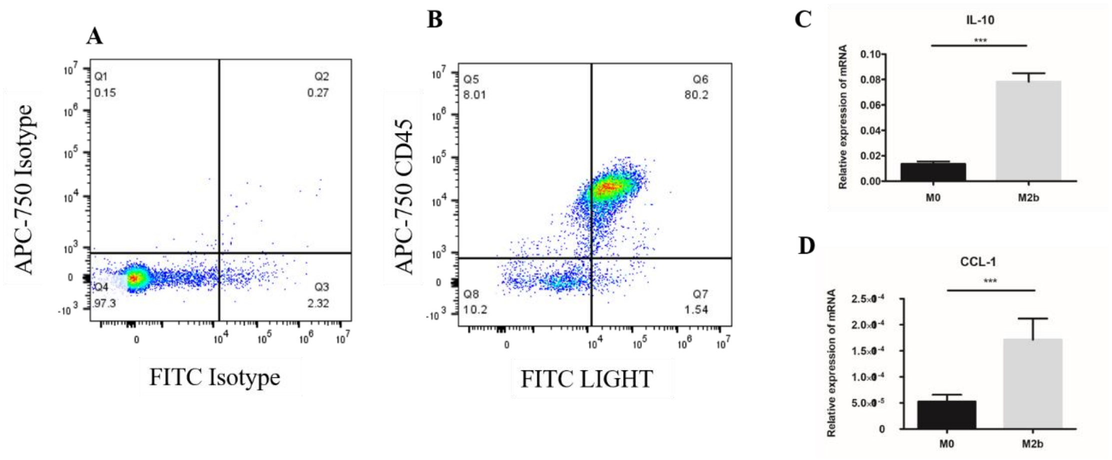

Supplement: S1 Fig — (A-B) M2b macrophages were subjected to staining for assessment of LIGHT and CD45 expression, followed by analysis through flow cytometry. More than 80% of the cells displayed both LIGHT and CD45 expression (n = 3). (C, D) qRT-PCR was employed to measure the mRNA levels of CCL-1 and IL-10 in macrophages. (n = 3 for each group). ***p < 0.001. “M0” indicates the M0 macrophage group, “M2b” indicates the M2b macrophage group. (PDF) [file pone.0288422.s001.pdf]
