# Supplementary figures and images for: M2b macrophages protect against doxorubicin induced cardiotoxicity via alternating autophagy in cardiomyocytes
Source: PLoS One. 2023 Jul 27;18(7):e0288422. doi: 10.1371/journal.pone.0288422 (PMC10374082; doi:10.1371/journal.pone.0288422)

**Figure S2 Images of the uncropped immunoblots shown in Fig. 3A**

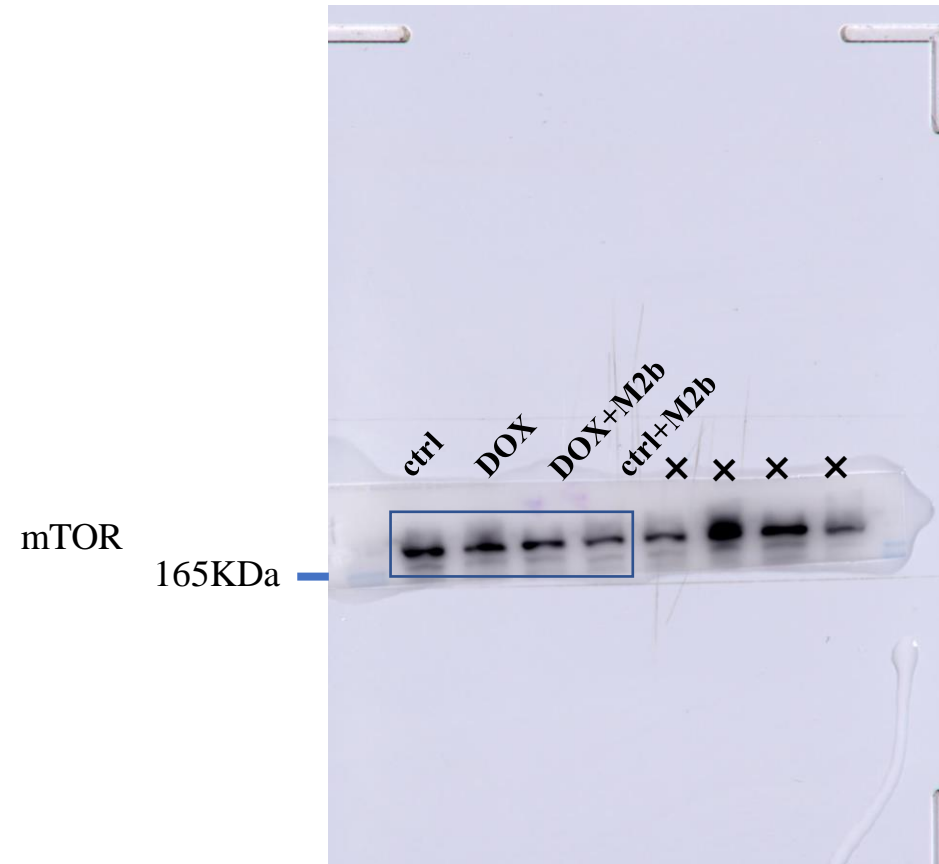

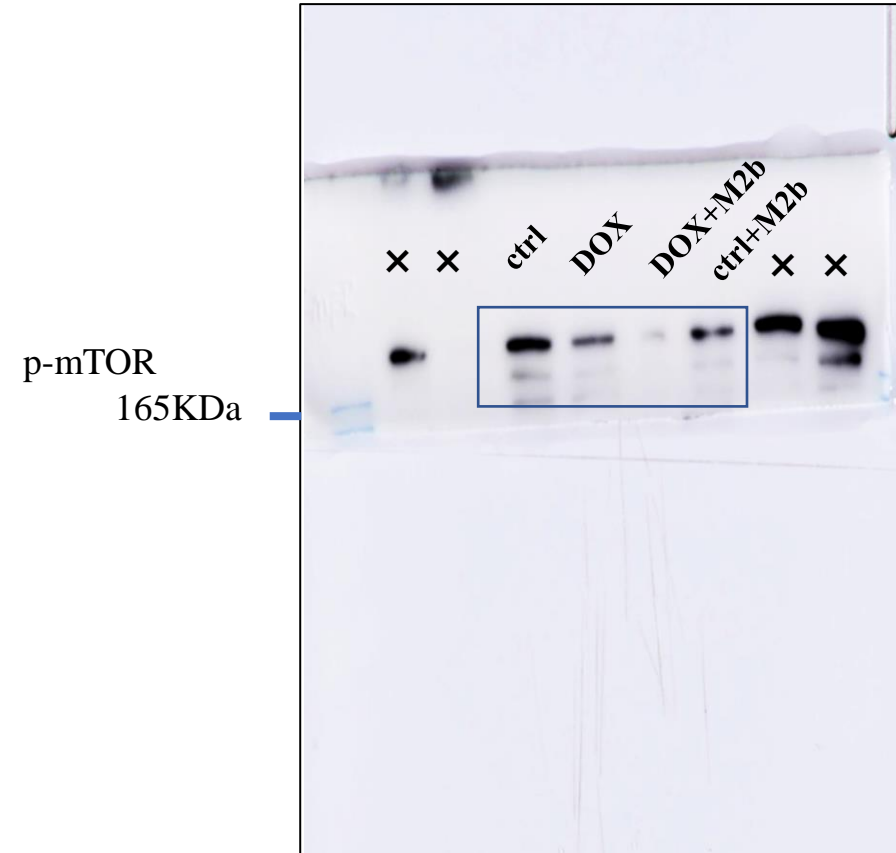

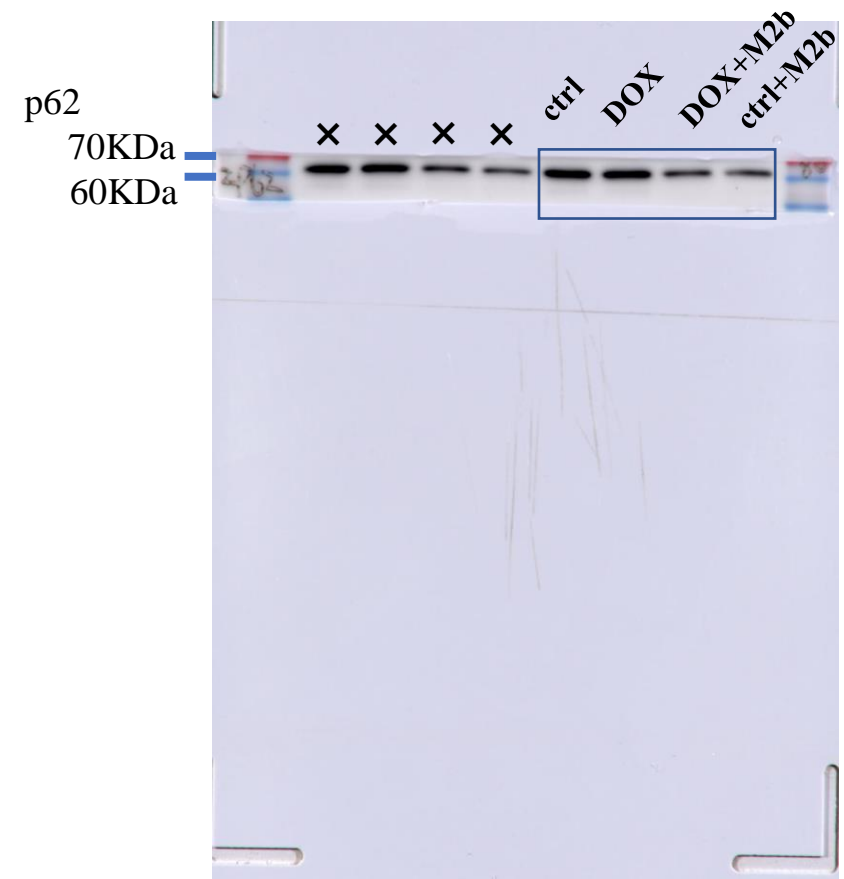

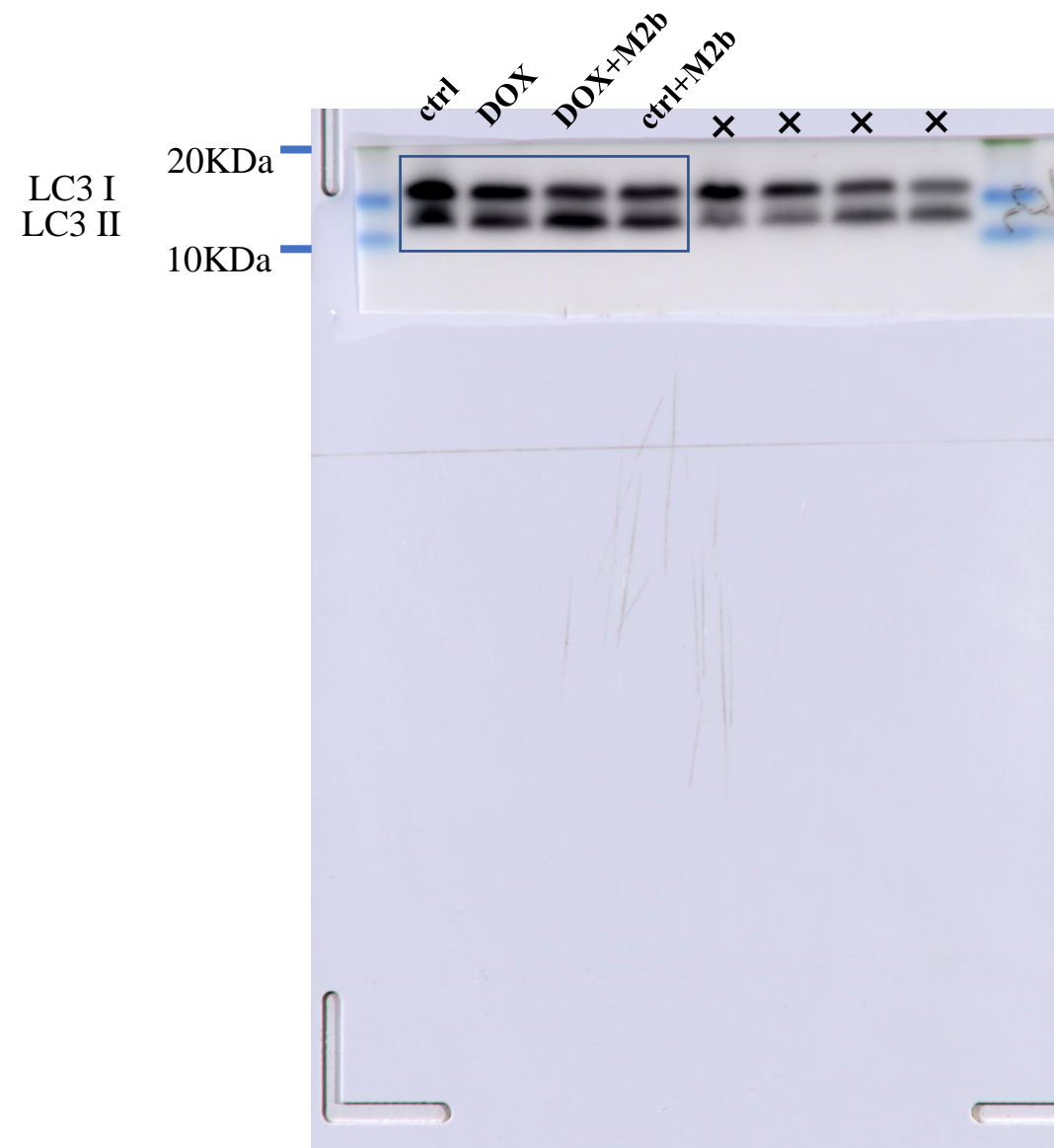

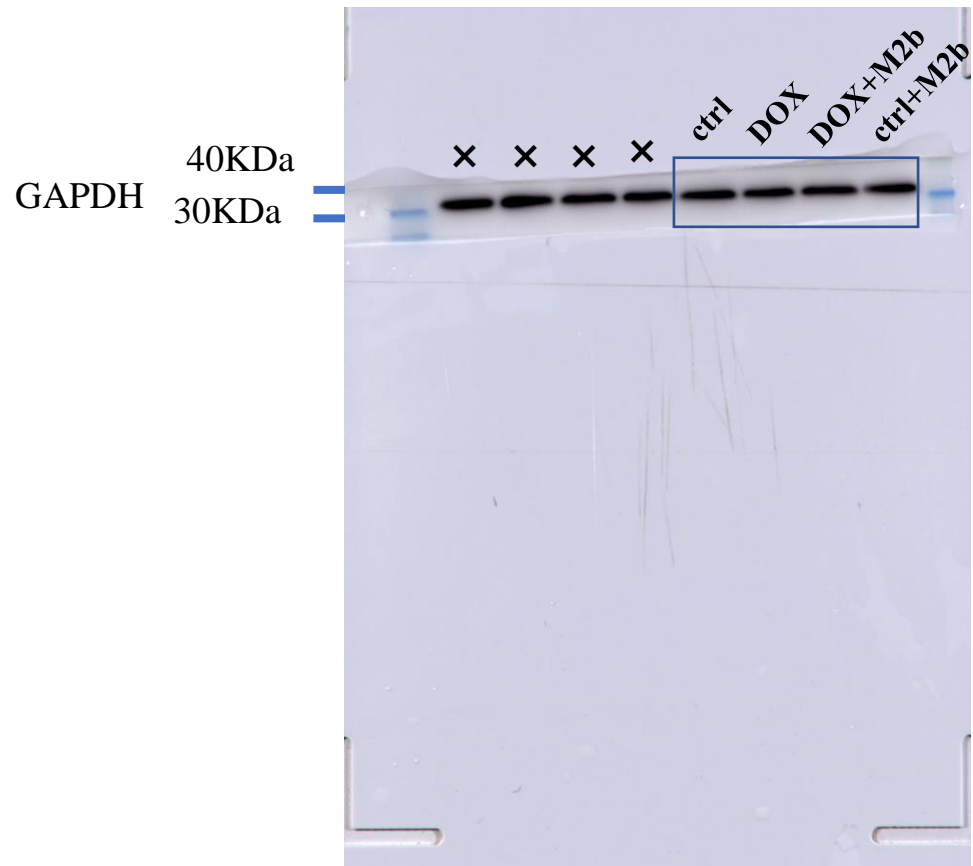

Supplement: S2 Fig — (PDF) [file pone.0288422.s002.pdf]

**Figure S3 Images of the uncropped immunoblots shown in Fig. 4A**

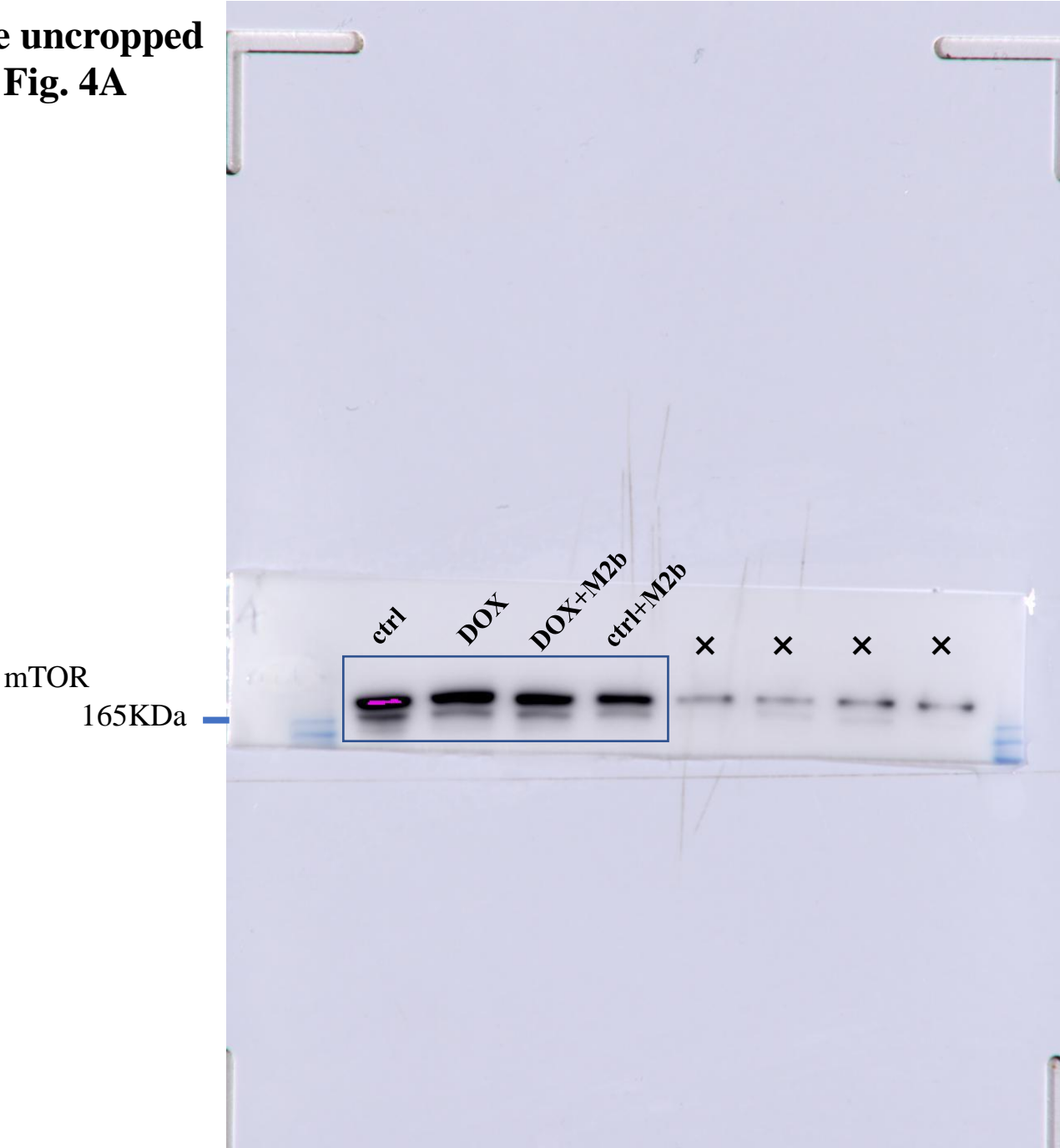

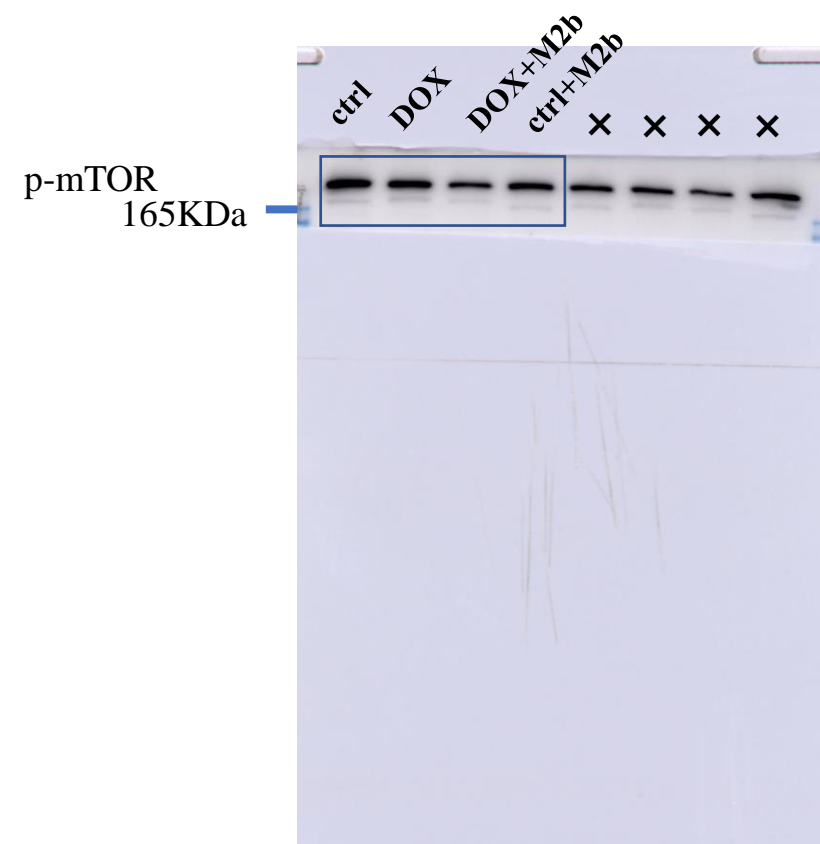

p62

70KDa

60KDa

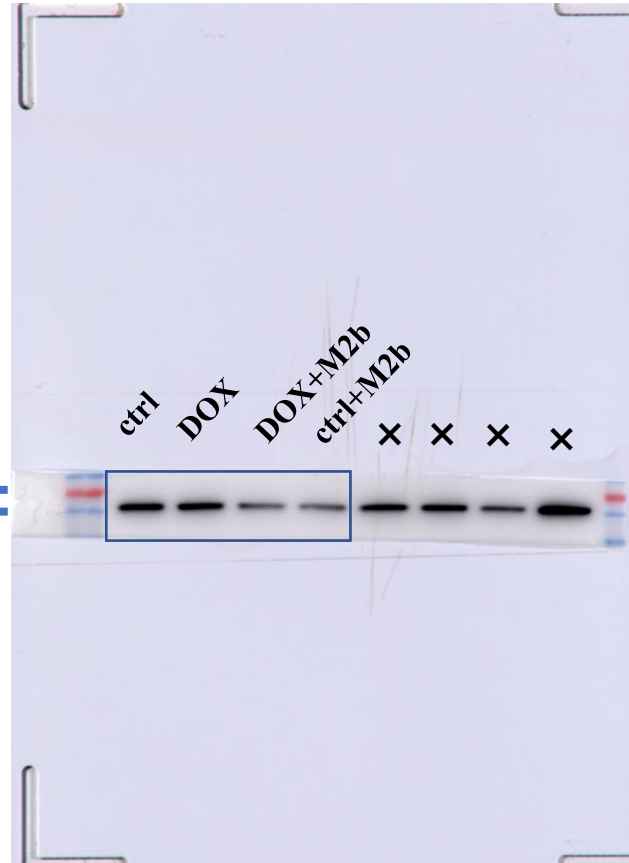

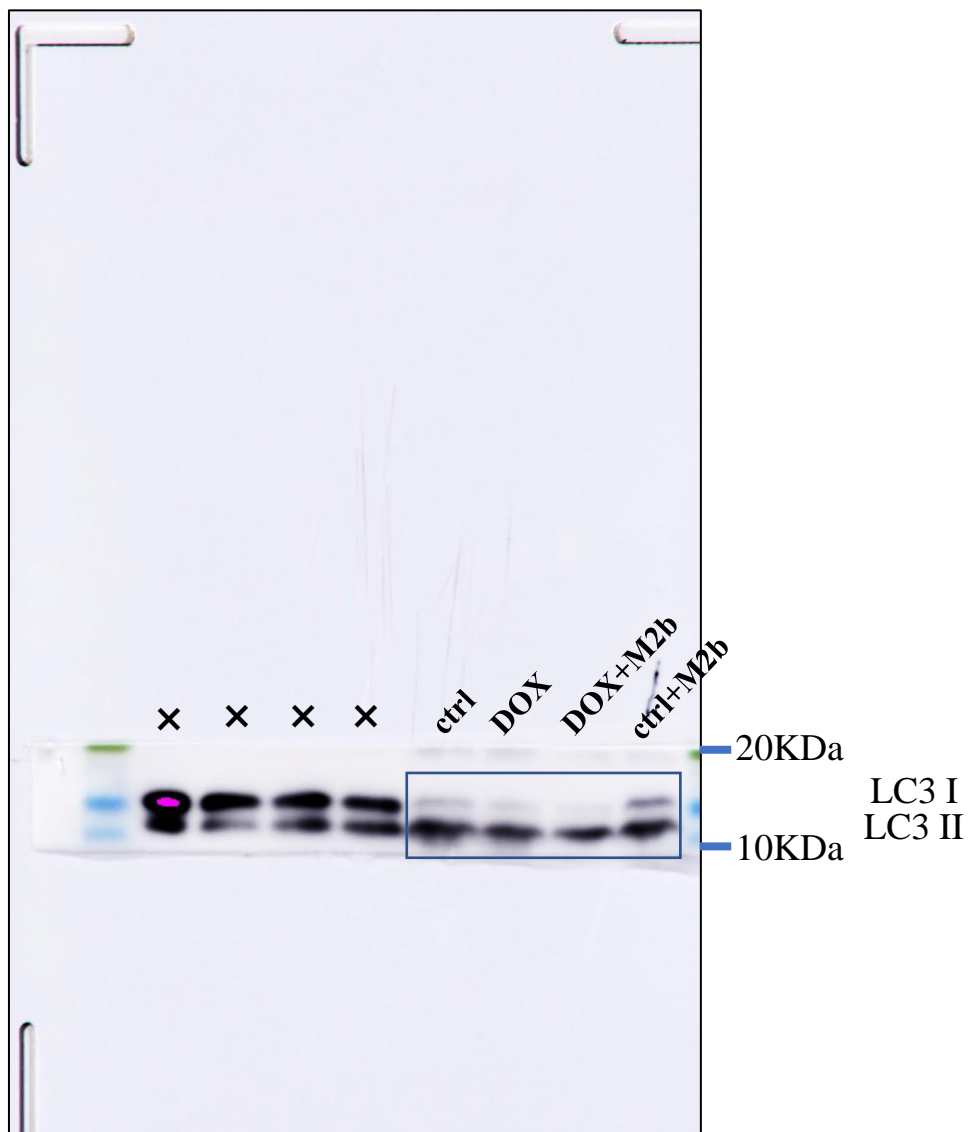

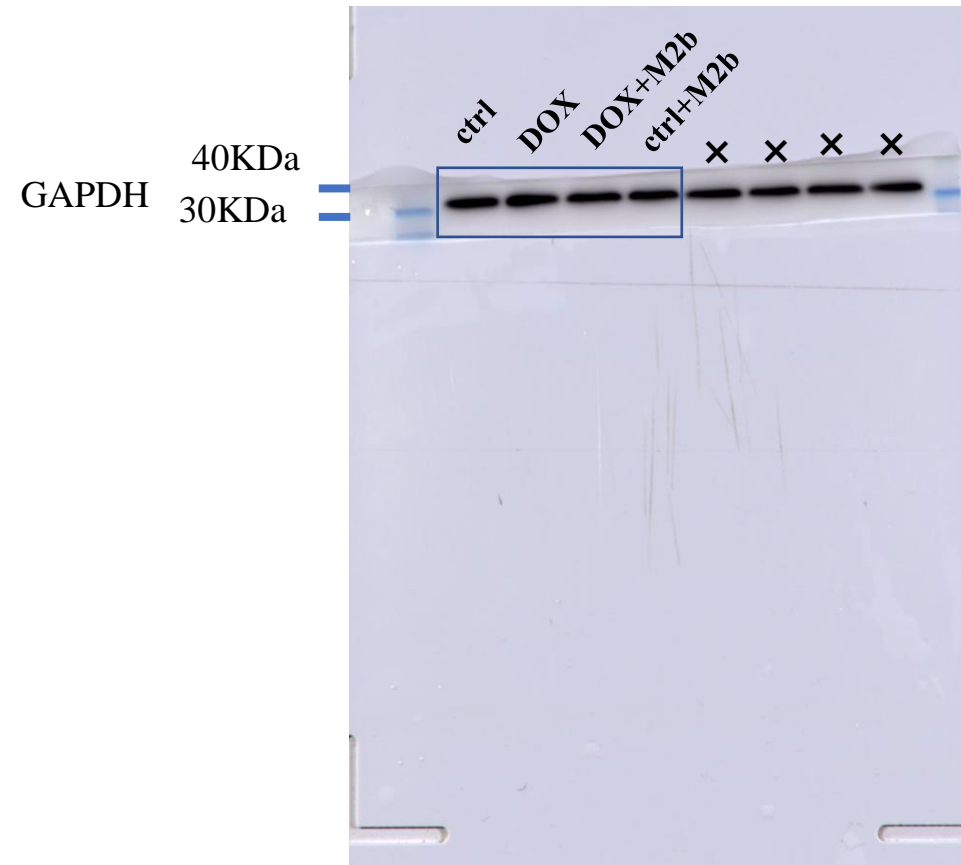

Supplement: S3 Fig — (PDF) [file pone.0288422.s003.pdf]
